# Determinants of household food security and maternal dietary diversity in rural Gedeo zone, southern Ethiopia: results from a cross-sectional study

**DOI:** 10.3389/fnut.2025.1523344

**Published:** 2025-07-09

**Authors:** Meskerem Jisso, Sibhatu Biadgilign, Amare Abera Tareke, Tizalegn Tesfaye, Tadesse Alemu Zerfu

**Affiliations:** ^1^Department of Public Health, College of Medicine and Health Sciences, Hawassa University, Hawassa, Ethiopia; ^2^PhD Fellow at Department of Food Technology, Safety and Health, Faculty of Bioscience Engineering, Ghent University, Ghent, Belgium; ^3^Independent Public Health Analyst and Research Consultant, Addis Ababa, Ethiopia; ^4^Department of Biomedical Sciences, College of Medicine and Health Science, Wollo University, Dessie, Ethiopia; ^5^College of Medicine and Health Sciences, Dilla University, Dilla, Ethiopia; ^6^International Food Policy Research Institute (FPRI), Addis Ababa, Ethiopia; ^7^Global Academy of Agriculture and Food Systems Royal (Dick) School of Veterinary Studies, University of Edinburgh (UoE)-Easter Bush Campus, Roslin, United Kingdom

**Keywords:** household food security, maternal dietary diversity, maternal nutrition, rural, Ethiopia

## Abstract

**Background:**

In Ethiopia, food insecurity and poor dietary diversity continue to affect maternal and child health, particularly in rural regions.

**Objective:**

We examined the status and determinants of household food security and maternal dietary diversity in rural Gedeo zone, southern Ethiopia.

**Methods:**

A cross-sectional study was conducted among randomly selected 422 households, and household food insecurity and women’s dietary diversity was measured. We conducted bivariable and multivariable logistic regression.

**Results:**

In this study, 65.5% of mothers (95% CI: 60.7–70.0%) and 27.9% (95% CI: 23.7–32.5%) of households had adequate dietary diversity and food security, respectively. Severe or moderate food insecurity predispose to inadequate dietary diversity, whereas, higher wealth status increased the odds of adequate dietary diversity. Maternal education increased the odds of adequate dietary diversity. Regarding household food insecurity, the size of the household having a member of 5–7 and 8–12 were 78 and 76% less likely to be food insecure among households compared to 1–4 members size [OR = 0.22 (95%CI: 0.07, 0.70)] and [OR = 0.24 (95% CI: 0.07, 0.83)] respectively. Those household having high in women’s dietary diversity were 83% less likely to have to food insecure among households compared to the lowest women’s dietary diversity [AOR = 0.17 (95% CI: 0.10, 0.31)].

**Conclusion:**

Household wealth status, maternal education, household food security status, pregnancy status were factors affected maternal dietary diversity. Family size and dietary diversity affected household food security status. Interventions should focus on maternal literacy, empowering women on income, assuring food security to increase maternal dietary diversity.

## Introduction

In low income countries, low-quality, monotonous diets are the norm and the risk for a variety of micronutrient deficiencies is high (1). Food security exists when all people, at all times, have physical and economic access to sufficient, safe, and nutritious food that meets their dietary needs and food preferences for an active and healthy life (2–4). Globally, malnutrition in all its forms also remains a challenge. The global prevalence of moderate or severe food insecurity has been slowly on the rise since 2014, the estimated increase in 2020 was equal to that of the previous 5 years combined (5). Not only does food insecurity in itself has deleterious effects on households and individuals but efforts at achieving food security may also pose a heavy economic toll if households must spend most of their income on obtaining food (6).

Nearly one in three people in the world (2.37 billion) did not have access to adequate food in 2020 – an increase of almost 320 million people in just 1 year (5). Close to 12 percent of the global population was severely food insecure in 2020, representing 928 million people—148 million more than in 2019 (5). In line with this, women of reproductive age living in resource-poor settings are at high risk of inadequate micronutrient intakes when diets lack diversity and are dominated by staple foods (1). The most recent estimates show that nearly 282 million people in Africa (about 20 percent of the population) were undernourished in 2022 and about 868 million people were moderately or severely food-insecure and more than one-third of them—342 million people—were severely food-insecure (7). According to the recent united nation report, majority of undernourished population have been found living in Asia (381 million) and more than 250 million live in Africa, there were nearly 144 million children under the age of five who suffered stunting, of which three quarters were found living in Southern Asia and sub-Saharan Africa, 47 million, i.e., 6.9% children under age of five were affected by wasting or acute under nutrition (8, 9). Dietary diversity (or dietary variety) refers to the number of foods consumed across and within food groups over a reference period, is widely recognized as being a key dimension and used as a proxy measure for food security, adequacy of energy/nutrient intake and diet quality (10–14). However, there is no consensus on the optimal standardized measure for dietary diversity (14). It also show the proxies of nutritional adequacy of the diet or as recommendations to maintain optimal health (10, 15).

Previous literatures indicate that dietary diversity indicators (DDIs) do not readily relate to health outcomes (11, 16). Minimum dietary diversity for women of reproductive age have paved the way for simple assessments of dietary diversity at the population level (11). Not only does food insecurity in itself has deleterious effects on households and individuals but efforts at achieving food security may also pose a heavy economic toll if households must spend most of their income on obtaining food (6). On a household level, presence of food insecurity probably suggests a high degree of vulnerability to a broad spectrum of consequences, including psychosocial dysfunction in children, socio-familial problems, and overall poor health status (6).

Measures of food security are pertinent where households are chronically vulnerable to deepening poverty, environmental and climatic shocks, rapid economic change, and conflict (17–20). As the country is designated as among the most famine-prone countries in Africa, has a long history of famines and food shortages, mostly due to climate shocks (21) and the population is vulnerable to weather fluctuations for its livelihoods and food security (22). Additionally, the latest evidence shows that, in the 2024 Global Hunger Index, Ethiopia ranks 102nd out of the 127 countries with a score of 26.2, Ethiopia has a level of hunger that is serious (23) and in the 2022 Global Food Security Index (GFSI), Ethiopia scored 44.5 out of 100 (24). Correspondingly, dietary diversity is a severe problem among the poor in the developing world, including Ethiopia (25). Similarly, the Gedeo zone faces significant challenges due to its fragile agroecology, high population density (one of the highest in the country), and persistent food insecurity issues. Empirical evidence for factors contributing to low minimum dietary diversity hardly exists (25–29) and different studies focused on the factors associated with minimum dietary diversity as well as household food security separately in different settings and there is limited evidence regarding the relationship between minimum dietary diversity and household food insecurity. Although studies have examined food insecurity and maternal nutrition in Ethiopia, little is known about how these factors interact at the household level in rural districts like Gedeo zone, where unique socioeconomic and agricultural conditions may influence dietary outcomes. The objective of the study was to examine the determinants of household food security and maternal dietary diversity in rural Gedeo zone, southern Ethiopia.

## Methods

### Study setting, design, and participants

The study was conducted in selected rural districts of the Gedeo zone, Southern Nations, Nationalities, and People’s Region (SNNPR), Ethiopia. The zone has six districts and two towns, namely, Dilla and Yirgachefe, each having between 24 and 40 kebeles, depending on geographic area and population. It is also one of the leading coffee producing areas in Ethiopia, supplying about 63% of the regional, and 28% of national coffee outputs annually. According to the 2007 national census, the zone has a total population size of 847,434 of which 424,742 were male and 422,692 were female whereas 739,653 were lived in rural area and 107,781 were lived in urban area (30). The study was conducted from February 1, 2019, to August 15, 2019. A community-based, cross-sectional study design was employed.

All mothers/caretakers (15–49 years) in the households of selected districts were eligible to partake in this study. Out of the six rural districts in Gedeo zone, two were drawn randomly using lottery method. A single population proportion formula, considering the following assumptions: 95% confidence level, 9.2% Proportion (P) for overall A-WEAI (Abbreviated Women’s Empowerment in Agriculture Index) A-WEAI, based on a similar study in rural Nepal (53), and margin of error of 2%. Adding in 10% for possible non-response rate and multiplying by 1.5 design effect, the total sample size required for the study was 428, but 422 households gave complete responses, yielding a response rate 98.6%. To ensure a representative sample of households, a stratified simple random sampling approach was used. First, they divided the study district, Wonago, into distinct urban and rural kebeles (lowest administrative units). Then, within each stratum (urban and rural), they randomly selected villages. Finally, households within these villages were chosen using simple random sampling, giving every household an equal chance of being included in the study. Households were the sampling unit for the study. Households who had lived for at least 6 months in the area were included in the study.

### Measures

#### Minimum dietary diversity for women

Women’s dietary diversity was measured using minimum dietary diversity of women (WDD-W) which was recently recommended by Food and Agriculture Organization of the United Nations (FAO) in 2016 (31). MDD-W is a dichotomous indicator of whether or not women have consumed at four out of seven defined food groups the previous day or night. According to the Food and Agricultural Organization, there are 16 groups of food. These food groups are cereals, vitamin A rich vegetables and tubers, white tubers and roots, dark green leafy vegetables, other vegetables, vitamin A riches fruit, other fruits, organ meat (liver, heart, kidney, etc.), flesh meat, eggs, fish and seafood, legumes (beans, peas, lentils, etc.), dairy products (milk and milk products), oils and fats, sweets and spices, condiments and beverages. Those women who reach this minimum in a population can be used as a proxy indicator for higher micronutrient adequacy, one important dimension of diet quality. The tool is validated through a multi-country study also found a strong association between dietary diversity and the micronutrient adequacy of the diet (1, 32).

Dietary diversity is therefore being increasingly adopted as a proxy indicator of micronutrient density or adequacy of the diet in large surveys and other data collection exercises (32). In this study, adequate dietary diversity represents women meeting the minimum level of dietary diversity. This means they have consumed ≥4-food groups during the previous day, whereas inadequate dietary diversity represents women who did not meet the minimum dietary diversity level. This means they have consumed <4 food groups during the previous day.

#### Household food security scale

Food insecurity was measured using the Household Food Insecurity Access Scale (HFIAS) developed by USAID’s Food and Nutrition Technical Assistance (FANTA) project, measure that reflects a household’s food security level for the previous month (33, 34).

The respondents were expected to answer these questions on behalf of all household members.

Household food security was assessed using the 9-item Household Food Insecurity Access Scale (HFIAS). Responses were scored on a scale of 0–3 and summed (range 0–27), and a higher HFIAS score indicated more food insecurity the household experienced (in terms of access to food). Households were categorized into four levels: food secure, mildly food insecure, moderately food insecure, and severely food insecure following HFIAS guidelines (35).

For analysis, these were dichotomized into food secure vs. food insecure. In this study, we used a combination of the three (mild, moderate, and severe) food insecure categories to form a dichotomous outcome variable (food secured and insecure). Food secured household refers if the respondent answers “No” to all, or “Yes” for only one of the eight standard questions of the Household Food Insecurity Experience Scale (HHFIES), while food insecure household If the respondent answers “Yes” to at least two of the questions of the HHFIES.

#### Data collection procedures

We used a pre-tested, structured, interviewer-administered questionnaire for collecting data and to interview mothers/caretakers. The questionnaire used for the survey includes socio-demographic variables, women’s dietary diversity scale, household food security scale, and other relevant information. The questionnaire was prepared in English and then translated to Amharic and Gedeufa languages, then back translated to English to maintain consistency. Trained enumerators (*n* = 12), after being trained for 3 days, were sent out to collect data. Questionnaires were extensively field-tested, revised, translated and back-translated to ensure data quality.

#### Statistical analyses

Data was entered, edited and cleaned using Statistical Package for the Social Sciences (SPSS) version 22 software packages and STATA 14 statistical software.

Descriptive statistics were calculated for socio-demographic characteristics and other parameters of interest for the participants. After dichotomizing both the dietary diversity and HHFIES, we conducted bivariable logistic regression to select candidate variables for multivariable logistic regression (cut point *p* < 0.25). In multivariable logistic regression, we reported adjusted odds ratio (AOR) and 95% confidence intervals (CI) to measure the effect. Statistical significance was declared using p value less than or equal to 0.05 in multivariable logistic regression.

#### Ethical consideration

Ethical approval was obtained from Dilla University College of Medicine and Health Science Institutional Review Board (IRB). Official co-operation letter was obtained from Gedeo zone health department and then submitted to respective district level offices. Investigators and all research assistants were trained in all ethical principles of the latest Helsinki declaration. Confidentiality, beneficence and privacy were cornerstones of the ethics aspect of this research.

## Results

### Socio-demographic characteristics of households

A total of 422 respondent were participated in this study. The mean age of the respondents was 32.05 years (standard deviation, SD ± 4.61). Among respondents 135(32%) and 259(61.37%) were less than 29 and 30–39 years age group, respectively. Majority of them were 383(90.76%) and 376 (89.10%) were ethnically Gedeo and follows protestant religion (Table 1).

**Table 1:** Socio-demographic and economics characteristics with household dietary diversity and household food security among households in rural Gedeo zone, southern Ethiopia.

Variables	Categories	Total sample n (%)	Food security status		*p*-value	Dietary diversity status		*p*-value
			Secure n (%)	Insecure n (%)		High n (%)	Low n (%)	
Mean age (± SD) in year		32.05 ± 4.61						
Maternal age (years)	Less than 29	135 (31.99)	34 (28.8)	101 (33.2)	0.681	59 (40.4)	76 (27.5)	0.026
	30–39	259 (61.37)	76 (64.4)	183 (60.20)		78 (53.4)	181 (53.4)	
	40–49	28(6.64)	8 (6.78)	20(6.60)		9 (6.16)	19 (6.16)	
Ethnicity of the mother	Gedeo	383 (90.76)	102 (86.4)	281 (92.4)	0.138	140 (95.9)	243 (88.0)	0.004
	Oromo	19 (4.50)	7 (5.93)	12 (3.95)		0 (0.00)	19 (6.88)	
	*Others	20 (4.74)	9 (7.63)	11 (3.62)		6 (4.11)	14 (5.07)	
Religion status of the mothers	Protestant	376 (89.10)	103 (87.3)	273 (89.8)	0.325	137 (93.8)	239 (86.6)	0.058
	Orthodox	43 (10.19)	15 (12.7)	28 (9.21)		9 (6.2)	34 (12.3)	
	Muslim	3(0.71)	0 (0.00)	3(0.99)		0 (0.00)	3 (1.09)	
Marital status of mothers	#Single	31 (7.35)	4 (3.39)	27 (8.88)	0.052	10 (6.85)	21 (7.6)	0.776
	Married	391 (92.65)	114 (96.6)	277 (91.1)		136 (93.1)	255 (92.4)	
Maternal educational status	Cannot read and write	200 (47.39)	49 (41.53)	151 (49.67)	0.440	69 (47.3)	131 (47.5)	0.010
	Can read and write only	100 (23.70)	31 (26.27)	69 (22.70)		23 (15.7)	77 (27.9)	
	Primary education	82 (19.43)	24 (20.34)	58 (19.08)		35 (24.0)	47 (17.0)	
	Secondary education and more	40 (9.48)	14 (11.86)	26 (8.55)		19 (13.0)	21 (7.61)	
Size of the household	1–4 members	38 (9.00)	4 (3.39)	34 (11.2)	0.023	15 (10.3)	23 (8.33)	0.802
	5–7 members	282 (66.82)	88 (74.58)	194 (63.8)		96 (65.7)	186 (67.4)	
	8–12 members	102 (24.17)	26 (22.0)	76 (25.0)		35 (24.0)	67 (24.3)	
Antenatal care (ANC) check-up during last pregnancy	<4 times	344(81.52)	96 (81.36)	248 (81.58)	0.958	110 (75.3)	234 (84.8)	0.017
	≥4 times	78 (18.48)	22 (18.64)	56 (18.42)		36 (24.7)	42 (15.2)	
Current pregnancy status	Yes	42(9.95)	9 (7.63)	33 (10.86)	0.320	23 (15.7)	19 (6.90)	0.004
	No	380 (90.05)	109 (92.37)	271 (89.14)		123 (93.3)	257 (93.1)	
Livestock density index	<=1	347 (82.23)	93 (78.81)	254 (83.55)	0.253	121 (82.9)	226 (81.9)	0.800
	2–3	75 (17.77)	25 (21.19)	50 (16.45)		25 (17.1)	50 (18.1)	
Household livestock ownership	No	218 (51.66)	55 (46.6)	163 (53.6)	0.196	89 (61.0)	129(46.7)	0.005
	Yes	204 (48.34)	63 (53.4)	141 (46.4)		57 (39.0)	147 (53.3)	
Household Wealth Status	Poorest	95 (22.51)	15 (12.7)	80 (26.3)	0.007	28 (19.2)	67 (24.3)	0.000
	Poorer/poor	79 (18.72)	32 (27.1)	47 (15.5)		43 (29.4)	36 (13.0)	
	Middle/Medium	84 (19.91)	21 (17.8)	63 (20.7)		31 (21.2)	53 (19.2)	
	Richer/Wealthy	80 (18.96)	24 (20.34)	56 (18.42)		16 (11.0)	64 (23.2)	
	Richest/Wealthiest	84 (19.91)	26 (22.03)	58 (19.08)		28 (19.2)	56 (20.3)	
Maternal body mass index (BMI) in kg/m^2^	Underweight (less than 18.5)	68 (16.11)	16 (13.56)	52 (17.1)	0.672	19 (13.0)	49 (17.7)	0.013
	Normal weight (18.5–24.9)	337 (79.86)	97 (82.2)	240 (78.9)		126 (86.3)	211 (76.5)	
	Overweight/obesity (greater than 25)	17 (4.03)	5 (4.24)	12 (3.95)		1 (0.70)	16 (5.80)	
Women’s dietary diversity (WDD)	Lowest	146 (34.60)	26 (22.03)	120 (39.47)	0.000			
	Medium	149 (35.31)	24 (20.34)	125 (41.12)				
	High	127 (30.09)	68 (57.63)	59 (19.41)				
Household Food Security Status	Food Secure	118 (27.96)				26 (17.8)	92 (33.3)	0.000
	Moderately FI	183 (43.36)				51 (34.9)	132 (47.8)	
	Severely FI	121 (28.67)				69 (47.3)	52 (18.8)	

*Others: Sidamo, Amhara and Wolayeta; Single: never married, divorced, separated, widowed.

### Dietary consumption pattern and composition of the food groups

**Figure 1:**
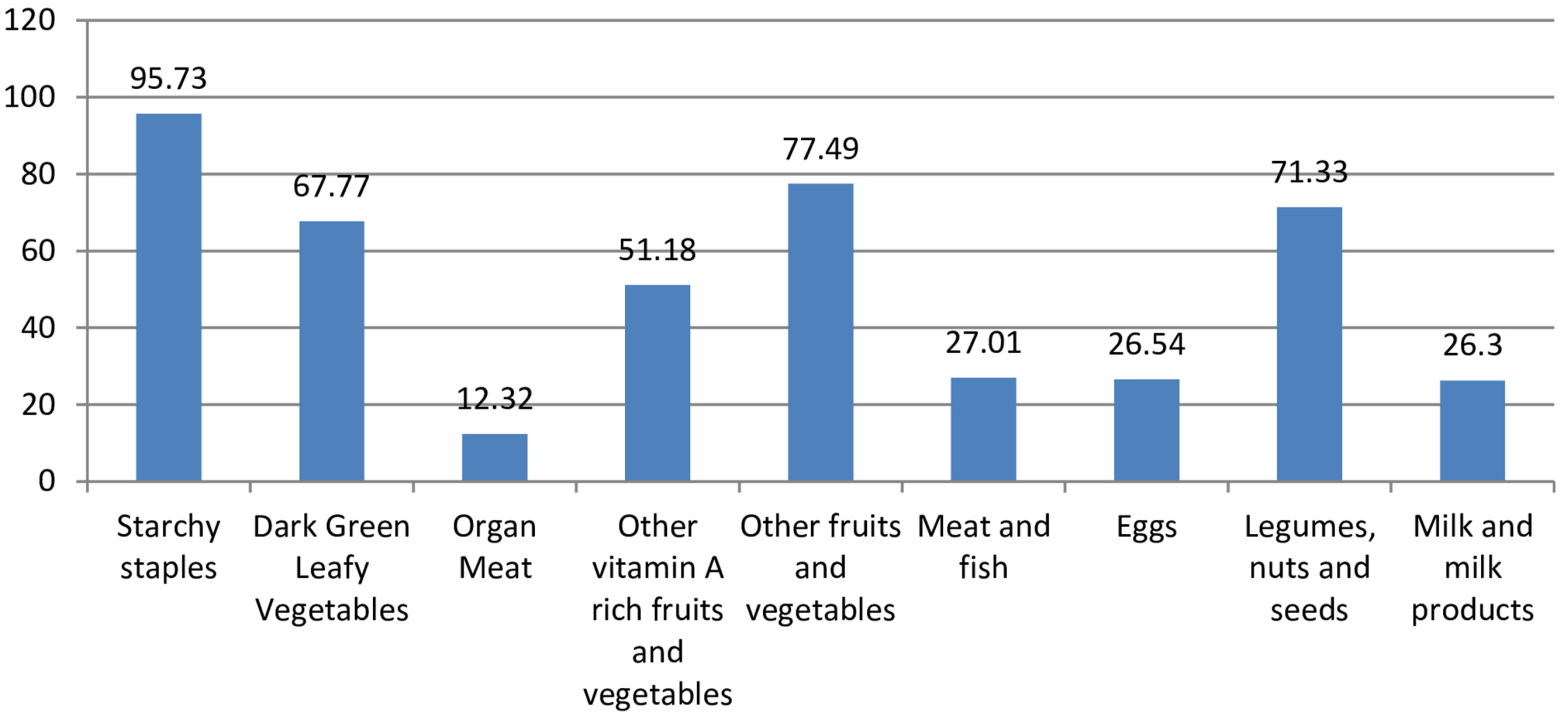
Proporion of food group consumed by the households in rural Gedeo, August 209.

Figure 1 demonstrate the proportion of food groups consumed by the households during the study period. The consumption of each food group was examined with household wealth status and the analysis indicate that a significant difference in the consumption of food groups were observed for organ meat, other fruits and vegetables, eggs, and milk and milk products in the studied households by their household wealth status. In addition to this, households food composition varied and have a difference category of food groups based on the household wealth status. In low wealth status category, the household consumed more on starchy staples (94.25%), Other fruits and vegetables (70.11%), dark Green Leafy Vegetables (62.07%) and other vitamin A rich fruits and vegetables (45.40%), respectively. Whereas, starchy staples (96.34%), other fruits and vegetables (86.59%),legumes, nuts and seeds (75.61%), and dark green leafy vegetables (73.78%) were the food groups consumed in a greater proportion among households with high wealth status category (Table 2). In this study, 65.5% of mothers (95% CI: 60.7–70.0%) and 27.9% (95% CI: 23.7–32.5%) of households had adequate dietary diversity and food security, respectively.

**Table 2:** Consumption of the food groups by household wealth status.

Food groups	Household wealth status						Pearson chi2	*p*-value
	Low		Medium		High			
	No.	%*	No.	%	No.	%		
Starchy staples	164	94.25	82	97.62	158	96.34	1.8139	0.404
Dark green leafy vegetables	108	62.07	57	67.86	121	73.78	5.3021	0.071
Organ meat	12	6.90	11	13.10	29	17.68	9.1497	0.010
Other vitamin A rich fruits and vegetables	79	45.40	44	52.38	93	56.71	4.3785	0.112
Other fruits and vegetables	122	70.11	63	75.00	142	86.59	13.5014	0.001
Meat and fish	43	24.71	21	25.00	50	30.49	1.6440	0.440
Eggs	30	17.24	20	23.81	62	37.80	18.7123	0.000
Legumes, nuts and seeds	118	67.82	59	70.24	124	75.61	2.5683	0.277
Milk and milk products	34	19.54	21	25.00	56	34.15	9.3834	0.009

No., number. *percentages indicate proportion of food groups consumed in each household wealth status.

### Determinants of dietary diversity

The study revealed that several factors were associated with dietary diversity. In this study, household having severely food insecure were 80% less likely to have a high diet diversity than those of food secure household [OR = 0.22 (95%CI: 0.12, 0.41)]. Interestingly, household having high wealth status had 2.52 times more likely to have high diet diversity as compared to low wealth status household [OR = 2.52 (95% CI: 1.31, 4.86)]. Those household having non-pregnant women in the household were 2.26 times more likely to have high diet diversity as compared to pregnant women in their household [OR = 2.26 (95% CI: 1.09, 4.68)]. Household having pregnant women following their antenatal care (ANC) check-up greater than or equal to 4 times during last pregnancy were 59% less likely to have high diet diversity as compared to pregnant women having check-up less than four times their household [OR = 0.41 (95% CI: 0.23, 0.74)]. Mother having an education level of secondary education and more 63% less likely to have a high diet diversity compared to those mothers having cannot read and write/no education level [OR = 0.37 (95% CI: 0.16, 0.84)] (Table 3).

**Table 3:** Bivariate and multivariable logistic regression analysis predicting the likelihood of having high household dietary diversity among households in rural Gedeo Zone, southern Ethiopia.

Variables	Crude	*P*-value	β	Adjusted	*P*-value
Maternal age (years)					
Less than 29	Ref			Ref	
30–39	1.80 (1.17, 2.77)	0.007	0.59	1.29 (0.79, 2.10)	0.303
40–49	1.64 (0.69, 3.88)	0.262	0.49	1.12 (0.42, 2.99)	0.813
Marital status of mothers					
#Single	Ref				
Married	0.89 (0.41, 1.95)	0.776	−0.11		
Maternal educational status					
Cannot read and write/No education	Ref			Ref	
Can read and write only	1.76 (1.02, 3.05)	0.043	0.57	1.52 (0.83, 2.781)	0.173
Primary education	0.71 (0.42, 1.20)	0.197	−0.35	0.68 (0.37, 1.25)	0.215
Secondary education and more	0.58 (0.29, 1.16)	0.122	−0.54	0.37 (0.16, 0.84)	0.017*
Size of the household					
1–4 members	Ref				
5–7 members	1.26 (0.63, 2.53)	0.510	0.23		
8–12 members	1.25 (0.58, 2.69)	0.571	0.22		
Antenatal care (ANC) check-up during last pregnancy					
<4 times	Ref			Ref	
≥4 times	0.55 (0.33, 0.90)	0.018	−0.60	0.41 (0.23, 0.74)	0.003*
Current pregnancy status					
Yes	Ref			Ref	
No	2.53 (1.33, 4.82)	0.005	0.93	2.26 (1.09, 4.68)	0.028*
Livestock density index					
≤1	Ref				
2–3	1.07 (0.63, 1.82)	0.800	0.07		
Household livestock ownership					
No	Ref			Ref	
Yes	1.78 (1.18, 2.67)	0.006	0.58	0.92 (0.53, 1.59)	0.770
Household wealth status					
Low	Ref			Ref	
Middle/Medium	1.18 (0.69, 2.01)	0.548	0.16	1.46 (0.76, 2.78)	0.252
High	1.88 (1.19, 2.97)	0.007	0.63	2.52 (1.31, 4.86)	0.006*
Household food security status					
Food Secure	Ref			Ref	
Moderately FI	0.73 (0.42, 1.26)	0.258	−0.31	0.63 (0.35, 1.12)	0.116
Severely FI	0.21 (0.12, 0.37)	0.000	−1.55	0.22 (0.12, 0.41)	0.000*
Maternal body mass index (BMI) in kg/m^2^					
Underweight (less than 18.5)	Ref			Ref	
Normal weight (18.5–24.9)	0.65 (0.37, 1.15)	0.140	−0.43	0.65 (0.34, 1.23)	0.186
Overweight/obesity (greater than 25)	6.20 (0.77, 50.1)	0.087	1.82	5.09 (0.58, 45.0)	0.143

*Significant predictors at *p* < 0.05, #Single includes never married, divorced, separated, widowed.

### Determinants of household food security

Table 4 shows that the determinants factors associated with household food security. In our study, the size of the household having a members of 5–7 and 8–12 were 78 and 76% less likely to be food insecure among households compared to 1–4 members size [OR = 0.22 (95%CI: 0.07, 0.70)] and [OR = 0.24 (95% CI: 0.07, 0.83)] respectively. Those household having high in women’s dietary diversity were 83% less likely to have to food insecure among households compared to lowest women’s dietary diversity [OR = 0.17 (95% CI: 0.10, 0.31)] (Table 4).

**Table 4:** Bivariate and multivariable logistic regression analysis predicting the likelihood of food insecure among households in rural Gedeo zone, southern Ethiopia.

Variables	Crude	*P*-value	β	Adjusted	*P*-value
Maternal age (years)					
Less than 29	Ref				
30–39	0.81 (0.50 1.30)	0.383	−0.21		
40–49	0.84 (0.34, 2.08)	0.709	−0.17		
Marital status of mothers					
#Single	Ref			Ref	
Married	0.36 (0.12, 1.05)	0.062	−1.02	0.59 (0.19, 1.82)	0.357
Maternal educational status					
Cannot read and write/No education	Ref			Ref	
Can read and write only	0.72 (0.42, 1.23)	0.231	−0.32	0.96 (0.53, 1.74)	0.893
Primary education	0.78 (0.44, 1.39)	0.407	−0.24	0.67 (0.35, 1.31)	0.245
Secondary education and more	0.60 (0.29, 1.24)	0.171	−0.51	0.48 (0.21, 1.08)	0.076
Size of the household					
1–4 members	Ref			Ref	
5–7 members	0.26 (0.09, 0.75)	0.013	−1.35	0.22 (0.07, 0.70)	0.010*
8–12 members	0.34 (0.11, 1.06)	0.064	−1.07	0.24 (0.07, 0.83)	0.024*
Antenatal care (ANC) check-up during last pregnancy					
<4 times	Ref				
≥4 times	0.98 (0.57, 1.70)	0.958	−0.01		
Current pregnancy status					
Yes	Ref				
No	0.68 (0.31, 1.46)	0.323	−0.39		
Livestock density index					
≤1	Ref			Ref	
2–3	0.73 (0.43, 1.25)	0.254	−0.31	1.35 (0.68, 2.69)	0.396
Household livestock ownership					
No	Ref			Ref	
Yes	0.75 (0.49, 1.16)	0.197	−0.28	0.89 (0.52, 1.52)	0.672
Household wealth status					
Low	Ref				
Middle/medium	1.11 (0.61, 2.01)	0.731	0.10		
High	0.84 (0.53, 1.35)	0.480	−0.17		
Women’s dietary diversity (WDD)					
Lowest	Ref			Ref	
Medium	1.13 (0.61, 2.07)	0.697	0.12	1.07 (0.57, 2.00)	0.835
High	0.19 (0.11, 0.32)	0.000	−1.67	0.17 (0.10, 0.31)	0.000*
Maternal body mass index (BMI) in kg/m^2^					
Underweight (less than 18.5)	Ref				
Normal weight (18.5–24.9)	0.76 (0.41, 1.40)	0.379	−0.27		
Overweight/obesity (greater than 25)	0.74 (0.23, 2.41)	0.616	−0.30		

*Significant predictors at *p* < 0.05, #Single includes never married, divorced, separated, widowed.

## Discussion

In the current study, maternal educational status, antenatal care check-up during last pregnancy, current pregnancy status, household wealth status and household food security status were significantly associated with likelihood of having high household dietary diversity, whereas size of the household and women’s dietary diversity were significantly associated with likelihood of food insecure.

In our study, the size of the household having a members of 5–7 and 8–12 were 78 and 76% less likely to be food insecure among households compared to 1–4 members size [OR = 0.22 (95%CI: 0.07, 0.70)] and [OR = 0.24 (95%CI: 0.07, 0.83)] respectively. From the body of literature, household size is a significant determinant of household food security. Large family size puts an extra burden on food consumption, and more likely to experience food insecurity in contrast to households with a small family size (36) as well as household budget for food is affected by household size, total earnings by household members and family structure in a given society (37). It was documented that pregnant women who have family size ≥ 5 were significantly associated with undernutrition in Gindeberet district, Oromia, Ethiopia (38). This is evidenced that as the household size increases, income per head decline and the less food secure the household becomes (36). Ina addition to this, women with large family sizes share meals (foods) with other family members (38).

Those household having high in women’s dietary diversity were 83% less likely to be food insecure households as compared to lowest women’s dietary diversity [OR = 0.17 (95%CI: 0.10, 0.31)]. The body of evidence showed that household socio-economic status (SES) is among the major contributing factors to the household food insecurity in Sub-Saharan Africa. Being of low SES, vis-à-vis low-income household status leads to the consumption of both an inadequate quantity and low-quality foods; the limited dietary diversity leads to a low-quality diet with poor vital nutrient content (39). Additionally, consumption of a poor quality diet, which is related to household food insecurity is associated with adverse health consequences such as obesity, chronic disease and nutritional disorders among children (40).

In this study, household having severely food insecure were 80% less likely to have a high diet diversity than those of food secure household [OR = 0.22 (95%CI: 0.12, 0.41)]. Similar to other evidence in other countries, dietary diversity practical to capture and useful indicators of food security status (10), maternal dietary diversity either during pregnancy or postnatal is decreased with household food insecurity (41) Dietary diversity is further significantly associated with nutrient adequacy which is an aspect of dietary quality for individuals (42, 43).

Other studies documented that household and individual dietary diversity are varied and influenced by food security (44–46). Interestingly, children from the food secure households had higher dietary diversity compared to the children from the food insecure households (44, 46, 47). This implies that household dietary diversity may therefore be an alternative easy pathway to estimate household food security (48).

Interestingly, household having high wealth status had 2.52 times more likely to have high diet diversity as compared to low wealth status household [OR = 2.52 (95% CI: 1.31, 4.86)]. There is a consistence evidence suggested that household wealth index can enhance and have positive effect on mother’s dietary variety (49–52). Additionally, there is a significant interaction observed between dietary diversity and household wealth index (53) as well as wealth indicators significantly and positively associate with dietary quality and robust determinants of dietary diversity (42). This is being associated with higher socioeconomic conditions (49). In rural Oromia region in Ethiopia, livestock ownership were more likely to attain the adequate dietary diversity (54). There is a positive associations between household assets and dietary diversity in Kenya (55), Ghana (56). The possible explanation could be household assets have been associated and used as a proxy indicator of the socioeconomic status of a household (55) and in developing countries have demonstrated that a DDS is associated with socio-economic status (56).

Those household having non-pregnant women in the household were 2.26 times more likely to have high diet diversity as compared to pregnant women in their household [OR = 2.26 (95% CI: 1.09, 4.68)]. Although pregnancy is a period of increased demand for food, and we expect increased consumption of food, the exact association of pregnancy and dietary diversity is unknown (57). Djossinou et al. in 2019, recruited mothers preconception and followed until pregnancy to evaluate the changes in dietary diversity, there were no change in maternal dietary diversity during pregnancy compared to the pre-pregnancy state (57). Another longitudinal study found no significant difference between diet during pregnancy and diet after weaning (58). Food avoidance (59) might contributed for lower dietary diversity during pregnancy, otherwise the concept needs further research.

Household having pregnant women following their antenatal care (ANC) check-up greater than or equal to 4 times during last pregnancy were 59% less likely to have high diet diversity as compared to pregnant women having check-up less than four times their household [OR = 0.41 (95% CI: 0.23, 0.74)]. In Western Ethiopia study, antenatal care follow-up was significantly associated with a higher probability of high dietary diversity score among pregnant women (60). In Northern Ghana study, frequency of ANC attendance was significant predictors of maternal DDS (56). high dietary diversity was associated with a lower level of antenatal stress or anxiety (54). Considering the association between dietary diversity and trimester of the pregnancy, the study in Kenya did not find any statistically significant association (55). In Ethiopian study, pregnant women who did not visit ANC were 2.52 times more likely to have inadequate dietary diversity than those who visit ANC during their pregnancy (38). This might be associated with the fact that nutrition education given to pregnant women at ANC sessions could contributed to increased diet diversity (56). While some of our findings may seem unexpected at first glance, we carefully consider potential reasons behind these patterns. For instance, the relationship between certain factors might actually work in reverse—women who have underlying health issues could be more likely to seek frequent antenatal care (ANC) yet still struggle with restricted diets due to their conditions. Additionally, even women with higher education levels might encounter economic hardships or cultural norms that limit their ability to access nutritious food, despite their knowledge. Lastly, we recognize that some observed associations could be influenced by measurement bias or residual confounding. Additionally, the inverse relationship between ANC visits and dietary diversity was unexpected. One possible explanation is that women with poorer nutritional status or complications may be more likely to attend ANC, thereby introducing reverse causation. Alternatively, the quality of nutrition education provided during ANC visits may be limited.

Mother having an education level of secondary education and more were 63% less likely to have a high diet diversity compared to those mothers having cannot read and write/no education level [OR = 0.37 (95% CI: 0.16, 0.84)]. In Islamabad, Pakistan study dietary diversity was not associated with sociodemographic, or socioeconomic status of pregnant women (61). Those pregnant women who had tertiary and secondary education had three times and two times more likely to achieve the adequate dietary diversity compared to those who had no formal education (54). Other studies also documented that education level among the pregnant women as the factors that were significantly associated with the minimum dietary diversity (55, 62). In a study conducted among women in Zimbabwe demonstrate that a linear and increasing association between dietary diversity and years of completed schooling. However, these results are primarily descriptive and not suggestive of any significant associations (50). Other studies also indicate that mother’s education and nutrition knowledge positively influenced their own dietary diversity (49, 63, 64). This is more attributable to the fact that educated women assign a significantly larger proportion of their household food budget to food groups that are nutritionally rich in micronutrients and mainly because of greater awareness and understanding of nutritional health benefits (62).

### Limitations of the study

This study despite the strengths, have the following limitations. First, this study relies on diet diversity as a proxy for assessing nutrient adequacy. Dietary diversity as a proxy to estimate micronutrient adequacy of the women lacks quantitative data. Dietary diversity does not provide precise, quantitative data, and it does not measure the exact nutrient intake, only infers it based on food variety. Second, another limitation of the study was the use of 24-h dietary recall to measure dietary diversity. This method has inherent limitation; eating habits significantly vary from day to day or season to season. While the 24-h dietary recall provides the snapshot of the current state, it may not represent the person’s usual diet or long-term trends. Third, since dietary data relies on women’s self-reported food consumption, inaccuracies may arise due to self-reporting bias, social desirability bias, or misrepresentation of actual intake, the MDD-W indicator focuses on food groups rather than quantities. This may result in overlooking nutritional adequacy and portion sizes. On the other hand, the binary (yes/no) nature of MDD-W may also overlook reasons behind dietary choices, such as cultural preferences or health restrictions. For example; if women consume small amounts from multiple food groups, they may meet MDD-W thresholds without achieving meaningful dietary diversity.

### Areas for future research

Building on the findings of this study, there are several areas, which warrant further investigation. Future research should investigate how cultural, regional, and socioeconomic factors influence both the effectiveness of MDD-W and food security across diverse populations. This will enable interventions to be more culturally sensitive and context specific. Moreover, research into the long-term effects of achieving MDD-W and food insecurity on pregnancy outcomes, maternal nutrition, and child growth and development is vital to the understanding of the broader implications of such nutritional measures. In addition, identifying key obstacles such as affordability, accessibility, gender norms that prevent women from consuming diverse diets; explore the need for additional or alternative indicators to better capture dietary quality as well as the use of biomarkers alongside self-reported dietary data for more accurate assessments.

## Conclusion

In conclusion, this study has shown that household wealth status, maternal education, household food security status, pregnancy status were factors affected maternal dietary diversity. On the other hand, family size and dietary diversity affected household food security status. Interventions should focus on maternal literacy, empowering women on income, assuring food security to increase maternal dietary diversity.

## Data Availability

The original contributions presented in the study are included in the article/supplementary material, further inquiries can be directed to the corresponding author.
